# Cardiovascular Organ Damage and Blood Pressure Levels Predict Adverse Events in Multiple Myeloma Patients Undergoing Carfilzomib Therapy

**DOI:** 10.3390/cancers11050622

**Published:** 2019-05-03

**Authors:** Giulia Bruno, Sara Bringhen, Ilaria Maffei, Andrea Iannaccone, Teresa Crea, Agnese Ravera, Anna Astarita, Fabrizio Vallelonga, Marco Salvini, Francesca Gay, Franco Veglio, Alberto Milan

**Affiliations:** 1 Hypertension Unit, Department of Medical Sciences, “Città della Salute e della Scienza” Hospital, 3-10126 Turin, Italy; giulia.bruno87@gmail.com (G.B.); ilaria.maffei@edu.unito.it (I.M.); iannaccone.andrea@gmail.com (A.I.); teresacrea@hotmail.it (T.C.); agnese.ravera@gmail.com (A.R.); astarita.unito@gmail.com (A.A.); vallelonga.fabrizio@gmail.com (F.V.); franco.veglio@unito.it (F.V.); 2 Myeloma Unit, Division of Haematology, University of Turin, “Città della Salute e della Scienza” Hospital, 3-10126 Turin, Italy; sara.bringhen@unito.it (S.B.); marco.salvini@unito.it (M.S.); fgay@cittadellasalute.to.it (F.G.)

**Keywords:** cardio-toxicity, multiple myeloma, arterial hypertension, cardiovascular organ damage, cardiovascular adverse event

## Abstract

Carfilzomib is a second-generation proteasome inhibitor approved for the treatment of multiple myeloma (MM). It seems to determine cardiovascular toxicity, primarily arterial hypertension. No predictive factors for cardiovascular adverse events (CVAEs) are known in patients affected by multiple myeloma treated with carfilzomib. We evaluated the role of cardiovascular organ damage parameters to predict CVAEs in MM patients taking carfilzomib. Seventy patients affected by MM were prospectively enrolled. A comprehensive cardiovascular evaluation was performed before carfilzomib therapy; they underwent a transthoracic echocardiogram and the assessment of carotid-femoral pulse wave velocity. All the patients were followed up (FU) to determine the incidence of CVAEs. The mean age was 60.3 ± 8.2, and 51% were male. The median FU was 9.3 (4.3; 20.4) months. A proportion of 33% experienced CVAEs, 91% of them had uncontrolled hypertension, 4.5% acute coronary syndrome, and 4.5% cardiac arrhythmias. Subjects with CVAEs after carfilzomib treatment had significantly higher blood pressure values, left ventricular mass (98 ± 23 vs. 85 ± 17 g/m^2^, *p* = 0.01), and pulse wave velocity (8.5 ± 1.7 vs. 7.5 ± 1.6 m/s, *p* = 0.02) at baseline evaluation compared to the others. Furthermore, baseline uncontrolled blood pressure, left ventricular hypertrophy, and pulse wave velocity ≥ 9 m/s were able to identify patients at higher risk of developing CVAEs during FU. These preliminary findings indicate that blood pressure control, left ventricular mass, and pulse wave velocity may predict CVAEs in MM patients treated with carfilzomib.

## 1. Introduction 

Carfilzomib is a second-generation proteasome inhibitor approved for the treatment of multiple myeloma (MM) that is demonstrated to improve the overall rate response and progression-free survival in MM patients compared to other chemotherapeutic strategies [1,2,3]. However, the incidence of cardiovascular adverse events (CVAEs) has been increasing since carfilzomib introduction: arterial hypertension and congestive heart failure, the most common CVAEs, are experienced by respectively 12% and 4% of MM patients treated with this new proteasome inhibitor [4,5]. The pathogenic mechanism underling carfilzomib-induced cardiovascular toxicity has not been clarified yet. However, myocardial cells seem to be sensitive to proteasome inhibitors because of the essential role of the ubiquitine-proteasome system for their intracellular metabolism [6]. Furthermore, proteasomal inhibition leads to the down regulation of eNOS (endothelial nitric oxide synthase) activity, which is responsible for decreased endothelial NO levels and thus development of arterial hypertension [7]. So far, no predictive factors for the development of cardiovascular toxicity have been identified in patients undergoing carfilzomib therapy [8].

Left ventricular mass is a morphological parameter that describes cardiac remodeling and the presence of left ventricular hypertrophy is a sign of cardiac organ damage [9]. Likewise, pulse wave velocity is the gold standard measurement of arterial stiffness and its use is suggested by current guidelines to determine vascular organ damage [9,10,11]. Left ventricular mass and pulse wave velocity are both independent predictors of CVAEs in the general population and specific subgroups [12,13,14,15]. Recent studies have suggested a correlation between left ventricular hypertrophy and the development of CVAEs during follow up in MM patients treated with the new proteasome inhibitor [16], but further analysis is required to define the potential predictive role of these parameters in this oncological population.

The aim of our study was to determine if parameters commonly used to assess cardiovascular organ damage are able to predict the incidence of CVAEs in MM patients undergoing treatment with carfilzomib.

## 2. Methods

From April 2017 to April 2018, 70 MM patients, followed by the Myeloma Unit (“Città della Saluta e della Scienza”, Turin), were prospectively enrolled. The inclusion criteria were the history of new diagnosed/refractory/relapsed MM in patients with clinical indication to carfilzomib treatment, and age ≥ 18 years. Patients were excluded if they had cardiac amyloidosis, were aged < 18 years, and denied informed consent.

The study protocol was approved by the ethic committee of our hospital “A.O.U. Città della Salute e della Scienza” of Turin (Protocol Number 0038655) and each patient signed a written consent form.

The enrolled patients underwent a comprehensive cardiovascular evaluation c/o our Echo Lab (Hypertension Unit, “Città della Salute e della Scienza”, Turin) before the beginning of carfilzomib infusions. We collected anamnestic information and performed a complete examination, including measurement of office blood pressure values, ambulatory blood pressure monitoring, electrocardiogram, transthoracic echocardiogram, and evaluation of carotid-femoral pulse wave velocity. Antihypertensive treatment was optimized in patients with uncontrolled blood pressure values, since office blood pressure < 140/90 mmHg was required to start every carfilzomib infusion.

Office blood pressure (BP) measurements were performed according to the current guidelines [9,17]. An automatic sphygmomanometer was used (Omron, M10-IT model). Three blood pressure values were assessed three consecutive times with measurements 1–2 min apart and the mean value was recorded and used for subsequent analysis. Optimal office blood pressure control was defined as the average BP < 140/90 mmHg.

Every patient underwent an ambulatory blood pressure monitoring (ABPM) according to the current recommendations [17]. A validated blood pressure measuring device (Takeda TM2430, A&D Company Ltd., Tokyo, Japan) was worn by the patients at the time of the visit and removed after a 24-h recording period. The device was set as to perform blood pressure measurements every 15 min for the entire 24 h period and the patients were asked to perform their usual daily activities during the exam. We obtained 24-h/day-time/night-time systolic, diastolic, and mean blood pressure values. Optimal ABPM blood pressure control was defined as the average 24-h BP < 130/80 mmHg, day-time BP < 135/85 mmHg, and night-time < 120/70 mmHg.

Every patient underwent a transthoracic echocardiogram (TTE) to evaluate cardiac organ damage. The exam was performed at rest with the patient lying in the left lateral decubitus position. Standard 2D-TTE images were acquired with an iE33 ultrasound machine (Philips Medical System, Andover, MA, USA) equipped with a sector probe (S5-1 transducer). Conventional parameters were assessed according to the current guidelines [18,19]. Left ventricular (LV) diameters and walls thickness were measured in parasternal long-axis view. LV geometry was defined by calculating left ventricular mass (LVM, obtained using the Deveraux formula indexed to both body surface area and height elevated 2.7) and relative wall thickness (RWT, obtained dividing the double of the LV inferolateral wall thickness by the LV internal diameter at end-diastole). Left ventricular hypertrophy (LVH) was diagnosed with an LV mass ≥115 g/m^2^ (≥49 g/m^2.7^) and ≥95 g/m^2^ (≥47 g/m^2.7^) respectively in men and women. LV volumes were assessed through the Simpson’s Biplane technique from apical 4- and 2-chamber views and indexed to body surface area, then used to evaluate LV systolic function as LV ejection fraction (EF). LV diastolic function was defined through the evaluation of early diastolic Tissue Doppler (TDI) velocities (e’ waves) of septal and lateral mitral annulus, mitral valve inflow (E and A wave velocity), tricuspid regurgitation peak velocity, left atrial volume (indexed to body surface area) and E/e’ ratio, according to the current recommendations [19]. Speckle tracking analysis was performed according to the current guidelines with a dedicated software (Automated Cardiac Motion Quantification, QLAB Cardiac Analysis, Philips, Andover, MA, USA): global longitudinal strain (GLS) was computed offline from standard 2D images of the LV in apical views (4-, 2-chamber, and long axis views) with manual adjustment of endocardial borders when needed following standardized protocols [20].

Carotid-femoral pulse wave velocity (cf-PWV) was measured to assess arterial stiffness. cf-PWV measurement was performed according to the current guidelines [11] with a validated instrument (Sphymocor system Atcor Medical, Sydney, Australia). A single applanation tonometer was used to obtain and record carotid and femoral pulse waveforms. The cf-PWV was calculated as the ratio between the distance covered by the waves and the time delay measured between the feet of the two waveforms. The mean of at least two cf-PWV measurements was considered for subsequent analysis.

### 2.1. Follow Up

The incidence of CVAEs during and after carfilzomib treatment was determined through periodic review of patient hematologic reports or phone calls.

CVAEs were assessed and graded according to the Common Terminology Criteria for Adverse Events version 5.0 (CTCAE 5.0) [21]: particularly we included arterial hypertension, heart failure, myocardial infarction, chest pain, dyspnea, arrhythmia, cardiac arrest, as previously reported in literature [5]. The “arterial hypertension” event referred to patients who developed arterial hypertension during carfilzomib treatment or hypertensive patients who had a rise in blood pressure levels during chemotherapy. Among these patients, we identified the ones who experienced an increase in blood pressure values requiring an intensification of the anti-hypertensive therapy before carfilzomib treatment and patients who had uncontrolled hypertension and needed a temporary interruption of carfilzomib infusions.

When feasible, a second clinical evaluation was planned after 6 months from carfilzomib treatment start to assess blood pressure control and optimize antihypertensive treatment if required.

### 2.2. Statistical Analysis

Statistical analysis was performed by using SPSS program (IBM SPSS Statistics, Version 22.0.0.0, IBM Corp., Armonk, NY, USA). The Kolmogorov Smirnov test was chosen to evaluate the distribution of the data. Quantitative variables were expressed as mean values and standard deviations or median values and interquantile ranges, according to their distribution. Qualitative variables were expressed as absolute values and percentages. Comparison between groups was performed with Student’s *t*-test and Chi-Square test for quantitative and qualitative variables, respectively. Kaplan Meier curves were obtained to investigate parameters associated with a higher risk of developing CVAEs. A *p*-value < 0.05 was assumed as level of statistical significance.

## 3. Results

The general characteristics of our cohort are shown in Table 1.

Mean age was 60.3 ± 8.2 years and 51.4% were male. In total, 37% of patients had a history of arterial hypertension. Other concurrent cardiovascular risk factors were obesity (31.4%), dyslipidemia (11.4%), diabetes (10%), and chronic renal failure (8.6%).

Mean MM duration was 4.3 ± 3.6 years. Most subjects (63, 90%) had relapsed or refractory MM and had already undergone chemotherapy with anthracyclines, immunomodulating agents, alkylanting agents and bortezomib. Median number of previous chemotherapeutic treatment lines was 2.5 (2;3). MM was mainly diagnosed at stage III according to the Durie-Salmon classification and stage I according to the International Staging System (ISS).

Mean office blood pressure (BP) and ABPM values were within normal limits (Table 2); however, 50% of patients did not have a baseline optimal blood pressure control and needed antihypertensive treatment introduction or adjustment.

TTE showed an average left ventricular mass within normal limits (90 ± 20 g/m^2^), with a prevalence of left ventricular hypertrophy (LVH) of 31.4%. Mean EF and GLS values were normal (EF 63 ± 7%, GLS −20.86 ± 2.34%). Parameters describing left ventricular diastolic function did not show any alteration, except for a mild reduction in septal and lateral e’ velocities. Finally, no vascular organ damage appeared in our cohort by evaluating cf-PWV (mean cf-PWV 7.8 ± 1.7 m/s).

Median follow up was 9.3 [4.3; 20.4] months. A total of 23 patients (32.9%) experienced CVAEs during follow up, within 3.6 [0.9; 6.1] months from carfizomib treatment start (Table 3): of these, 17 (74%) experienced increase in blood pressure values to > 140/90 mmHg requiring antihypertensive treatment intensification, 4 (17%) had uncontrolled blood pressure values requiring a temporary interruption of carfizomib treatment; finally, 1 patient (4.5%) had a non-ST-elevated myocardial infarction and 1 (4.5%) developed atrial fibrillation. According to the CTCAE 5.0 severity classification [20], most CVAEs were grade 1–2 (18, 78%), only 22% (5 events) were grade ≥ 3.

**Figure 1:**
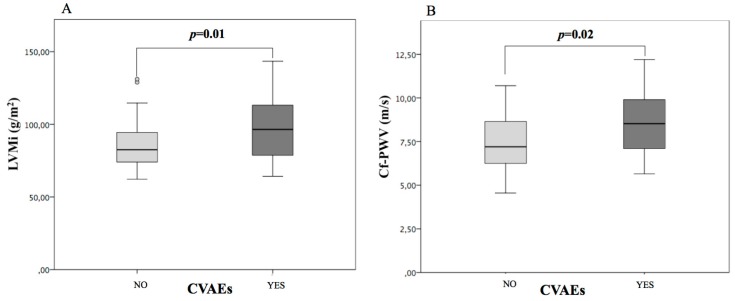
Cardiovascular organ damage in patients with and without cardiovascular adverse events during follow up. LVMi = left ventricular mass indexed to body surface area (**A**); cf-PWV = carotid-femoral pulse wave velocity (**B**). CVAEs = cardiovascular adverse events.

We divided our population into 2 groups based on the incidence of CVAEs during follow up (Table 4). No significant differences in age, sex, anthropometric variables, traditional cardiovascular risk factors, MM characteristics (duration, previous treatments, total carfilzomib dose) were seen between groups. However, baseline blood pressure control was significantly worse in patients who experienced CVAEs. Cardiovascular organ damage was significantly different, too (Figure 1): left ventricular mass and the prevalence of left ventricular hypertrophy were higher in the group of subjects with CVAEs (LVMi 98 ± 23 vs. 85 ± 17 g/m2, *p* = 0.01; LVH 52.2% vs. 21.7%, *p* = 0.01); furthermore, cf-PWV was higher in patients with CVAEs (8.5 ± 1.7 m/s vs. 7.5 ± 1.6 m/s, *p* = 0.02). However, no differences in baseline GLS values were seen between groups. Blood pressure control and cardiovascular organ damage were similar in patients who had grade 1–2 and grade ≥ 3 CVAEs.

Likewise, we observed that patients with baseline LVH and cfPWV ≥ 9 m/s had an increased incidence of CVAEs compared to those who had normal LVMi and cf-PWV < 9 m/s at first visit (55% vs. 23%, *p* = 0.003, and 53% vs. 25%, *p* = 0.02, respectively). Cf-PWV = 9 m/s was chosen as the most sensitive cut-off by creating a ROC curve. Similarly, CVAEs rate was significantly higher when baseline BP was uncontrolled (47% vs. 20%, *p* = 0.046).

**Figure 2:**
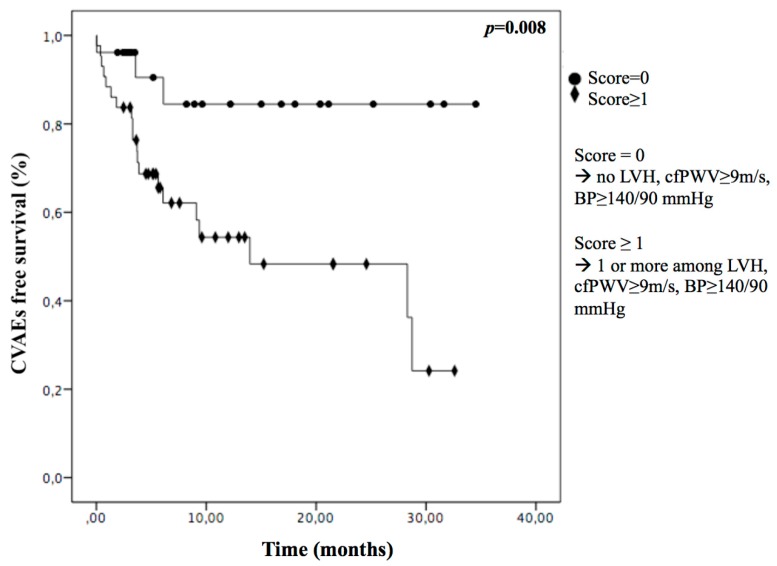
Kaplan Meier curves in multiple myeloma (MM) patients with (score ≥ 1) or without (score = 0) baseline uncontrolled hypertension, left ventricular hypertrophy or cf-PWV ≥ 9 m/s. LVH = left ventricular hypertrophy; cf-PWV = pulse wave velocity; BP = blood pressure.

Finally, we found the presence of at least one of these conditions (LVH, cf-PW ≥ 9 m/s, BP ≥ 140/90 mmHg) was sensitive in recognizing who would experience CVAEs (Figure 2): we assigned a score to each patient based on the presence (score = 1) or absence (score = 0) of each parameter and we saw a significantly lower incidence of CVAEs in patients with a score = 0 (12%) compared to patients with a score ≥ 1 (47%), *p* = 0.008.

**Figure 3:**
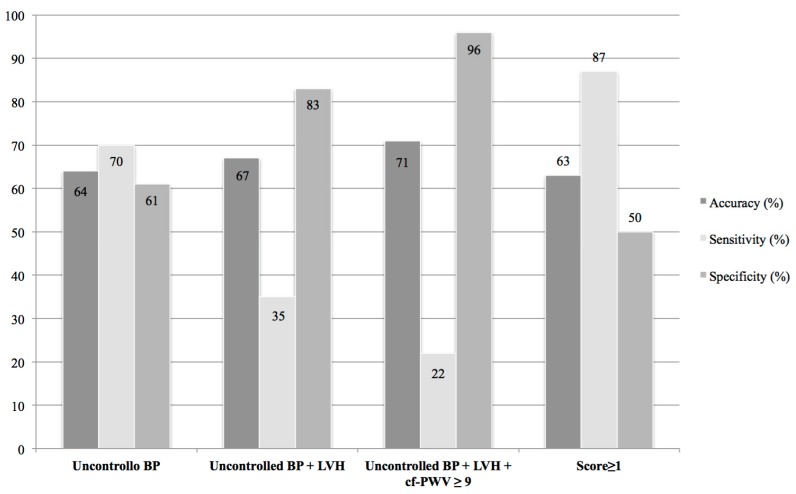
Sensitivity, specificity and accuracy of clinical and cardiovascular parameters. BP = blood pressure; LVH = left ventricular hypertrophy; cfPWV = pulse wave velocity; Score ≥ 1 = patients with 1 or more among BP ≥ 140/90, LVH, cf-PWV ≥ 9 m/s.

BP control represented the most sensitive parameter in recognizing patients at risk of developing CVAEs (BP sensitivity 70%), while LVH and cf-PWV allowed us to reach a higher specificity and to be more accurate. Furthermore, the score ≥ 1 was even more sensitive than BP control alone and enabled us to identify patients that could develop adverse events after carfilzomib treatment with a sensitivity of 87% and the same accuracy (63%) as BP control alone (Figure 3).

Finally, 48 patients underwent a follow up clinical evaluation that was performed after a mean period of 6 ± 2.6 months at our Echo Lab. The examination was primarily based on checking office blood pressure values and optimizing the antihypertensive treatment if necessary.

Anthropometric variables did not change from the first visit. We observed an improvement in blood pressure control: systolic and diastolic office blood pressure values were significantly lower compared to the first evaluation (SBP 126 ± 14 mmHg vs. 136 ± 19 mmHg, *p* = 0.001; DBP 72 ± 9 mmHg versus 78 ± 11 mmHg, *p* = 0.002) and the prevalence of patients with office blood pressure < 140/90 raised from 43% to 68% (*p* = 0.02). Patients treated with antihypertensive drugs were 66% (versus 46% at baseline visit), with a greater number of medications per person.

## 4. Discussion

To the best of our knowledge, this is the first study aimed at evaluating the prognostic value of parameters commonly used to determine cardiovascular organ damage in predicting the incidence of cardiovascular adverse events in MM patients treated with carfilzomib.

In our cohort, the baseline clinical characteristics did not highlight significant differences between patients that did and did not experience CVAEs during follow up, except for office blood pressure control, which was optimized before carfilzomib start (BP < 140/90 mmHg was necessary to begin the infusions). However, in our population, baseline left ventricular mass and cf-PWV were significantly higher in patients who experienced CVEAs during carfilzomib therapy. Furthermore, we recognized left ventricular hypertrophy, cf-PWV ≥ 9 m/s, and baseline uncontrolled office blood pressure as parameters able to distinguish subjects with increased risk of developing CVAEs over time.

Left ventricular hypertrophy is known to be a direct consequence of an elevated afterload; several studies have demonstrated its independent predictive value on incidence of CVAEs over time [12]. Carfilzomib is thought to determine a direct cardiac damage: myocardial cells are sensitive to proteasome inhibitors because of the important role of the ubiquitine-proteasome system for their metabolism; the inhibition of proteasome activity leads to myocardiocyte apoptosis [22]. Moreover, carfilzomib-induced endothelial dysfunction causes arteriolar vasoconstriction because of reduced NO levels [7]. Clinically, this could translate into a tendency to develop left ventricular remodeling and LVH, as recently suggested in a longitudinal study on a cohort of MM patients treated with carfilzomib [23]. According to this evidence, MM patients with baseline cardiac organ damage, particularly LVH, could be predisposed to develop CVAEs after treatment with carfilzomib, which is known to be cardio-toxic and likely involved in additional cardiac remodeling.

The ubiquitine-proteasome system plays an important role in endothelial cells, too: the inhibition of proteasome activity is associated with increased intracellular oxidative stress, reduced eNOS activity [7], and accelerated vascular aging, characterized by arterial stiffness and atherosclerosis [24]. Previous studies have demonstrated that chemotherapeutic drugs, like anti-VEGF, cause arterial hypertension through the reduction of endothelial NO levels and subsequent increase in peripheral resistance, with a rise in cf-PWV during treatment [25].

A recent animal study [26] has also proved a spasmogenic effect of carfilzomib: after pretreatment with this proteasome inhibitor, rabbit aortic strips had an increased basal tone compared to the untreated ones and showed enhanced response to vasopressors and reduced vasodilatatory response to vasodilator agents. These data might explain why patients with baseline increased arterial stiffness and higher cf-PWV values might be predisposed to have CVAEs during carfilzomib treatment: the rise in blood pressure levels, the most frequently reported adverse effect, could be determined by an additional endothelial dysfunction and an increased carfilzomib-induced vascular tone on already damaged and stiff vessels.

Left ventricular hypertrophy and cf-PWV are known predictors of CVAEs incidence in different populations [12,27,28]. These parameters could probably be useful in the oncological population as well [29,30], to help stratify their risk of experiencing cardiovascular adverse effects during chemotherapy.

However, the results obtained in our 70-patient cohort are preliminary and need to be confirmed by further investigation on a larger population of MM patients, in order to state with stronger evidence the potential predictive value of such parameters.

As regards cardiovascular adverse events, in our population the incidence was 32.9%, but only 7.2% of patients had a grade ≥ 3 CVAE. In the overall cohort, 30% had arterial hypertension, and only 5.7% required a temporary interruption of chemotherapy. These data overlap with what has been previously demonstrated in literature [5]. ASPIRE, ENDEAVOR, and FOCUS studies showed an incidence of arterial hypertension of 11%–32% in patients treated with carfilzomib compared to those that underwent different chemotherapeutic strategies [1,2,3].

The incidences of myocardial infarction and arrhythmias (1.4%) were similar to the ones described in the literature [4]. Furthermore, we did not see any cases of heart failure, which usually follows carfilzomib treatment in 4% of cases [4]. The low incidence of major CVAEs with the need of stopping chemotherapy encourages the use of this new proteasome inhibitor.

Finally, patients who underwent a follow up evaluation better blood pressure control then during their first visit: this is probably related to the optimization of the antihypertensive treatment.

Our intervention is likely to have determined a reduced incidence of major events in our cohort: CVAEs after carfilzomib treatment in carefully followed patients from a cardiovascular health standpoint can be limited to a mild–moderate worsening in blood pressure, allowing patients to continue the oncological therapy.

The main limitation of this study is represented by the small cohort. However, it is important to underline that this is the first study with the aim of systematically evaluating the cardiovascular profile of MM patients undergoing carfilzomib treatment, in order to find predictors of adverse events following chemotherapy. The second important limitation of the study is the short-term follow up. Further evaluations are needed to determine the incidence of long-term CVAEs.

## 5. Conclusions

Our study demonstrated that a comprehensive cardiovascular evaluation and management in patients undergoing carfilzomib treatment may help identify subjects at higher risk of experiencing CVAEs during follow up and determine a low incidence of major CVAEs, which are mainly represented by worsening in blood pressure control, necessitating the interruption of chemotherapy.

The results we obtained indicate that the presence of at least one among LVH, high cf-PWV, and uncontrolled hypertension, before carfilzomib infusions, may have a predictive role in identifying patients who are likely to experience CVAEs during treatment. Although BP control has proved to be more sensitive than LVH and cf-PWV, performing a TTE and measuring cf-PWV can improve our ability to correctly identify patients at risk of CVAEs compared to blood pressure control alone.

These findings are preliminary. If additional investigation on a larger cohort confirms the primary results, these parameters may be included in the baseline evaluation of MM patients undergoing carfilzomib treatment, helping differentiate subjects who need a more aggressive antihypertensive treatment and a stricter follow up from the beginning.

## Figures and Tables

**Table 1:** General characteristics, cardiovascular risk factors, and oncological history.

Variable	Population *n* = 70
Age, years	60.3 ± 8.2
Male sex, *n* (%)	36 (51.4)
Weight, Kg	73.3 ± 15.2
Height, cm	163 ± 11
BSA, m^2^	1.78 ± 0.22
BMI, Kg/m^2^	27.6 ± 4.7
**Cardiovascular risk factors**	
Arterial hypertension, *n* (%)	26 (37.1)
Obesity, *n* (%)	22 (31.4)
Coronary artery disease, *n* (%)	2 (2.9)
Diabetes, *n* (%)	7 (10)
Chronic renal failure, *n* (%)	6 (8.6)
Dyslipidemia, *n* (%)	8 (11.4)
Active smoking/previous smoking, *n* (%)	5 (7.1)/24 (34.3)
**Oncological history**	
MM duration, years	4.3 ± 3.6
Relapsed/Refractory MM, *n* (%)	63 (90)
Previous therapy	
Antracyclines, *n* (%)	26 (37.1)
Alkylating agents, *n* (%)	59 (84.3)
Immunomodulating agents, *n* (%)	42 (60)
Bortezomib, *n* (%)	56 (80)
MM staging	
DS: stage I-I–III (%)	9.1-27.3–63.6
ISS: stage I-I–III (%)	53.5-30.2–16.3
Total carfilzomib dose, mg/m^2^	665 [295; 1 082]

* Quantitative values are expressed as mean ± SD or median [interquantile range]. BSA = body surface area; BMI = body mass index; MM = multiple myeloma; DS = Durie-Salmon classification; ISS = International Staging System.

**Table 2:** Office blood pressure and ambulatory blood pressure monitoring (ABPM).

Office Blood Pressure	Population (*n* = 70)
Office BP, mmHg	131 ± 18 / 77 ± 11
Office BP < 140/90 mmHg, *n* (%)	35 (50)
Antihypertensive drugs, *n*	1 [1;2]
**ABPM**	
24 h SBP, mmHg	120 ± 11
24 h DBP, mmHg	71 ± 7
24 h MBP, mmHg	88 ± 8
24 h HR, bpm	77 ± 13
24 h SD, mmHg	14 ± 5
Day-time SBP, mmHg	124 ± 12
Day-time DBP, mmHg	75 ± 8
Day-time MBP, mmHg	92 ± 8
Day-time HR, bpm	80 ± 14
Day-time SD, mmHg	13 ± 6
Night-time SBP, mmHg	111 ± 13
Night-time DBP, mmHg	64 ± 7
Night-time MBP, mmHg	80 ± 9
Night-time HR, bpm	70 ± 13
Night-time SD, mmHg	10 ± 4
Dipping, %	11 ± 7

* Quantitative values are expressed as mean ± SD or median [interquantile range]; ABPM = ambulatory blood pressure monitoring; SBP = systolic blood pressure; DBP = diastolic blood pressure; MBP = mean blood pressure; HR = heart rate; SD = standard deviation.

**Table 3:** Cardiovascular adverse events during and after carfilzomib treatment.

Event	All Events	Severe Events, Severity Score ≥ 3 *
Arterial hypertension	21 (30)	4 (5.6)
- requiring intensification of antihypertensive therapy during carfilzomib treatment, *n* (%)	17 (24.3)	2 (2.8)
- requiring a temporary interruption in carfilzomib infusions, *n* (%)	4 (5.7)	2 (2.8)
Heart failure, *n* (%)	0 (0)	0 (0)
Myocardial infarction, *n* (%)	1 (1.4)	1 (1.4)
Chest pain, *n* (%)	0 (0)	0 (0)
Dyspnea, *n* (%)	0 (0)	0 (0)
Arrhythmias, *n* (%)	1 (1.4)	0 (0)
Valvular heart disease, *n* (%)	0 (0)	0 (0)
Pulmonary hypertension, *n* (%)	0 (0)	0 (0)
Thromboembolic events, *n* (%)	0 (0)	0 (0)
Cardiac arrest, *n* (%)	0 (0)	0 (0)
Total events, *n* (%)	23 (32.9)	5 (7.2)

* Defined according to CTCAE 5.0 (Common Terminology Criteria for Adverse Events).

**Table 4:** Comparison between patients with and without cardiovascular adverse events (CVAEs) during follow up.

General Characteristics and Comorbidities	CVAE− (*n* = 47)	CVAE+ (*n* = 23)	*p* Value
Male sex, *n* (%)	24 (51.1)	12 (52.2)	0.93
Age, year	60.4 ± 7.8	60.1 ± 9.1	0.91
BMI, Kg/m^2^	27.2 ± 4.4	28.5 ± 5.1	0.25
Arterial hypertension, *n* (%)	16 (34)	10 (43.5)	0.44
Diabetes, *n* (%)	4 (8.5)	3 (13)	0.52
Dyslipidemia, *n* (%)	4 (8.5)	4 (17.4)	0.27
Chronic renal failure, *n* (%)	4 (8.5)	2 (8.7)	0.98
**Multiple myeloma**			
Total carfilzomib dose, mg/m^2^	708 (428; 1108)	540 (103; 1118)	0.19
**Office Blood pressure and ABMP**			
SBP, mmHg	127 ± 18	141 ± 19	0.03
DBP, mmHg	74 ± 12	82 ± 8	0.09
Office BP < 140/90 mmHg, *n* (%)	28 (59.6)	7 (30.4)	0.02
24 h SBP, mmHg	118 ± 12	124 ± 9	0.04
24 h DBP, mmHg	71 ± 7	74 ± 7	0.10
**Cardiovascular organ damage**			
LVMi, g/m^2^	85 ± 17	98 ± 23	0.01
LVH, %	10 (21.7)	12 (52.2)	0.01
EF, %	63 ± 7	62 ± 8	0.65
LAVi, mL/m^2^	30 ± 9	28 ± 9	0.32
TDI e’, cm/s	8.0 ± 1.7	7.1 ± 1.4	0.04
E/e’	8.7 ± 2.4	9.3 ± 3.0	0.38
GLS, %	−20.96 ± 2.08	−20.72 ± 2.72	0.74
cf-PWV, m/s	7.5 ± 1.6	8.5 ± 1.7	0.02

* Quantitative values are expressed as mean ± SD or median [interquantile range]; BMI = body mass index; SBP = systolic blood pressure; DBP = diastolic blood pressure; LVMi = left ventricular mass indexed to body surface area; LVH = left ventricular hypertrophy; EF = ejection fraction; LAVi = left atrial volume indexed to body surface area; E = transmitral Doppler E wave velocity; TDI = Tissue Doppler Imaging; e’ = TDI e’ wave velocity; GLS = global longitudinal strain; cf-PWV = carotid-femoral pulse wave velocity.
